# ASO Author Reflections: Hepatobiliary Surgeons are Spurred to Implement Totally Minimally Invasive Techniques for Perihilar Cholangiocarcinoma Surgery

**DOI:** 10.1245/s10434-020-09188-w

**Published:** 2020-10-01

**Authors:** Robert Sucher, Daniel Seehofer

**Affiliations:** Department of Visceral, Transplant, Thoracic and Vascular Surgery, University Clinic Leipzig, Leipzig, Germany

## Past

The ongoing revolution in laparoscopic liver surgery has delivered scientific evidence of improved short-term outcomes and uncompromised long-term oncologic results for patients with resectable malignant liver disease, including cholangiocarcinoma (CCA) cases.^1^ Technically demanding surgical steps, such as radical lymphadenectomy and extrahepatic bile duct resection with biliary reconstruction, are mandatory in patients with perihilar CCA (pCCA). However, minimally invasive pCCA surgery remained the last garrison, currently intended to be stormed by a plethora of specialized hepatobiliary surgeons.^2^

## Present

In the article by Sucher et al., we share our strategy of a total laparoscopic approach for pCCA type 3b resection. The hepaticojejunostomy, in our opinion the most challenging part of the procedure, which is still commonly performed through a larger service incision at the end of a minimally invasive liver resection, even by highly decorated hepato-pancreato-biliary (HPB) surgical teams, was furthermore intended to be performed using a minimally invasive approach. To accomplish our goal, we successfully applied a ‘parachute’ running suture technique for biliary-enteric reconstruction, which facilitated an improved view on both components of the anastomosis, the posterior wall of the hepatic duct, and the corresponding jejunal segment. Additionally, short biliary drains were placed, aiming to splint the anastomosis and hence prevent cholestasis in the early postoperative period. Using the laparoscopic approach for pCCA surgery, hepatobiliary surgeons are urged not to compromise on radical lymphadenectomy, which has recently been shown to be equally effective and accurate when compared with open surgery.^3^

## Future

Every new technology that brings advantages also comes with inconveniences. In the case of laparoscopic pCCA surgery, a standardized procedure for intraoperative bile leakage testing is still in demand. Enhanced intraoperative visualization techniques such as near infrared indocyanine green staining methods^4^,^5^ and hyperspectral imaging^6^ might add substantial benefits to the identification of not only vascular structures but also biliary structures. In our opinion, these novel technologies might well be exploited for the specific detection of biliary leaks in future laparoscopic liver surgery.
